# In search of a Rohingya digital diaspora: virtual togetherness, collective identities and political mobilisation

**DOI:** 10.1057/s41599-023-01553-w

**Published:** 2023-02-15

**Authors:** Anas Ansar, Abu Faisal Md. Khaled

**Affiliations:** 1 Bonn Center for Dependency and Slavery Studies (BCDSS), University of Bonn, Bonn, Germany; 2 Department of International Relations, Bangladesh University of Professionals (BUP), Dhaka, Bangladesh

**Keywords:** Cultural and media studies, Complex networks

## Abstract

Frequently called the most persecuted minority in the world, the Rohingyas have suffered systematic violence and oppression in Myanmar since the 1970s. Today, the vast majority of the nearly three million Rohingyas are in exile, escaping state-sponsored human rights violations and persecution in the Rakhine state of Myanmar—a place they call “home”. Neighbouring Bangladesh, which currently hosts over a million displaced Rohingya, has been a ‘sanctuary’ for at least the last four decades. A sizable community has also emerged successively in other South-East Asian countries and pockets of Australia, Europe and North America. In this context, bringing together issues at the crossroads of (im)mobilities, online connectivity and the quest for identity, this study examines the role of social media platforms in forming and shaping new types of diaspora activism among the exiled Rohingyas. Drawing on yearlong online ethnographic findings, it unpacks how digital platforms constitute a space for togetherness, where diasporic Rohingya identities are constructed, contested and mediated. Analysing recurring themes and patterns of engagement on these web-based platforms, the paper looks at how diasporic civic and political e-activisms are transforming the very contours of Rohingya identity formation and their pursuit of recognition. Finally, focusing on such a creative constellation of socio-cultural and political issues in virtual space, we demonstrate how Rohingyas practice a politics of resistance and recognition when confronting the policy pretensions of Myanmar’s government.

## Introduction

*“In the first place, we do not like to be called ‘refugees.’“*—Hannah Arendt.

In her classic essay *“We Refugees”*, Hannah Arendt describes the endless cognitive anxiety amongst the Jews of Europe as they fled the continent and made a new life in exile (Arendt, 2017). She depicts how difficult it is to relate to the psychological effects of political non-existence unless one has traversed the liminal space of a refugee. The contemporaneous of forced displacement, statelessness and the relentless search for a ‘safe place’ and an ‘identity’ across the globe reminds us how recurring and prescient Hannah Arendt’s century-old observation remains. Set within such interconnected trajectories of violence, statelessness and an endless search for identity, this paper puts a spotlight on Myanmar’s displaced Rohingyas—a scattered community in the process of becoming a nascent diaspora as a result of their protracted displacement. Since 2017, after their mass exodus from the Rakhine state into neighbouring Bangladesh, exiled Rohingya communities have started highlighting their plight while asserting a distinct ethnic identity (Ansar & Khaled, 2022; Abraham & Jaehn, 2020). Considering their increasing involvement in social, cultural and political issues on social media platforms, this article explores how the Rohingya diaspora has coalesced in digital spaces to build a transnational identity and how their digital activism has evolved to include a political dimension over time.

Frequently termed ‘the world’s most persecuted minority’, the Rohingyas have been subjected to persistent human rights violations, including ethnic cleansing, statelessness and possibly even genocide (Khaled, 2021; Ansar, 2020; Ibrahim, 2018; Alam, 2018). By introducing punitive policies, Rohingyas have been categorically denied a range of fundamental rights by the Myanmar government, including the freedom of movement, rights to education, primary health facilities, having family, marriage and employment (Ansar & Khaled, 2021; Uddin, 2020). Ethnic cleansing and persecution of Rohingyas in Myanmar and denial of their citizenship (therefore, effectively rendering them stateless) has been the political strategy of the successive military regimes. Today, the vast majority of the nearly three million Rohingyas is displaced, mostly in neighbouring Bangladesh, Malaysia, India and Thailand, as well as in in pockets across Europe, Australia and North America.

The predicament of the Rohingyas essentially remain unresolved in exile. The ambiguity around Rohingya’ s legal status pertaining to their perceived statelessness, irregular migration and lack of comprehensive protection policies in the host countries add to their struggle to survive and sustain. Most Rohingya-hosting Asian countries deny their rights as refugees stipulated in the 1951 Refugee Convention. Their confinement in makeshift settlements and sprawling camps, ambiguous and/or undocumented legal status and host countries’ arbitrary practices create certain mobility constraints, which Aziz (2022, p. 1) refers to as “immobility turn” or limited mobility within “situations of unequal power”. Furthermore, mobility is linked to legality and capacity in modern nation-states, which Rohingyas lack in Myanmar due to draconian military laws banning social gatherings and community mobilisation (Ansar & Khaled, 2022, p. 281). In exile, this “arrested refugee mobilities” (Hoffstaedter, 2019) that the Rohingya community continues to endure produces both horizontal (i.e., spatial/geographic) immobility and vertical (social) immobility, which cyclically compound each other (Jernigan, 2019). Nevertheless, amid such challenges, a diaspora network has grown, especially since 2017, with considerable online imprints. We define the growing digital footprint of the Rohingya community as the emergence of a ‘Rohingya Digital Diaspora’. Highlighting their increasing online participation, our findings reveal how such engagement reinvigorates a collective identity, mobilises civic resistance and builds a virtual ‘community of hope’ by providing material and emotional support.

Reflecting on these evolving Rohingya online engagements, this study makes a threefold contribution to digital diaspora studies. First, we examined how the (re)production of Rohingya identities on social media demonstrates their hybrid, multi-layered and fluid nature. Second, considering the constrained offline space and (im)mobility dynamics, we looked into how access to social media can yield an opportunity for ethnic and religious minorities such as Rohingyas for transnational lobbying, advocacy and agenda-framing towards building a strategic and positive consensus around their cause. Third, while celebrating “digital optimism”, a nuanced reflection on the offline inequalities, such as those manifested by age, gender, internet access, economic status and spatiality, needs to be adequately contextualised.

## The debate on Rohingya identity: the unfolding of belonging, exclusion and exile

The nation-state centric identity has always been marked by a high degree of hybridity and ambiguity in post-colonial societies. In South and Southeast Asia, “questions surrounding nationality, citizenship, religion and identity are recurrent themes between the countries once united but separate nation-states now” (Sengupta, 2020, p. 114). Similarly, ethnic and religious identity and space are constantly being contested, refined and reorganised in the political landscape of Myanmar. This is particularly prominent in the bordering Rakhine state, where the formation of Rohingya identity has been heavily influenced by such fluidity (Ansar, 2020, p. 4).

Several issues appear to be decisive when we explore the documentation and broad historical analysis of how questions of Rohingya identity and conflict in the Rakhine state have arrived at this stage. These include: the stripping of the Rohingya citizenship and their statelessness (Uddin, 2020; Holliday, 2014); the role of Rohingyas during the colonial period (Alam, 2018; Ibrahim, 2018); military dictatorship and the emergence of *Taing-Yin-tha* meaning “national races” (Cheesman, 2017); and religion and the perceived threat from Islam (Ansar, 2020; Kyaw, 2015; Wade, 2017). These are just some of the profound issues to unpack in order to understand the making of the current crisis.

Broadly, three lines of arguments can be identified when exploring the Rohingya identity. First, some scholars claim a historic Rohingya presence in Myanmar (Uddin, 2020; Shafie, 2019; Ibrahim, 2018). Secondly, there are scholars who tend to discredit such narratives that argue Rohingya is a post-colonial political identity promoted by the Muslim political elites in Arakan as a tool to promote their fight for political autonomy after the Second World War (Leider, 2018; Tonkin, 2014). The third line of argument instead takes a critical approach between the two opposing narratives. Going beyond the polarising opinions, it argues that the fundamental question of the process of identity formation and the complex status of the ethnic and religious minorities in post-colonial nation-state formation should be in the spotlight (Ansar, 2020; Sengupta, 2020; Alam, 2018).

One of the watershed moments in modern-day Myanmar’s identity politics is the emergence of *Taing-Yin-tha*, or “the indigenous races”, under the 1982 citizenship law introduced by the military dictatorship in Myanmar. The concept of *Taing-Yin-tha* emerged as a decisive political language that provides the guideline of which facts are accepted and rejected in determining membership in Myanmar’s political community. In contemporary Myanmar, *Taing-Yin-tha* has become an exemplary term of state: a contrivance for political inclusion and exclusion, political eligibility and domination (Cheesman, 2017, p. 462). The Rohingya were not included among the 135 official indigenous races. Consequently, some 2.5 million Rohingyas are excluded from *Taing-Yin-tha*, making them one of the world’s largest stateless populations. They remain the only community in independent Myanmar whose citizenship is “still unresolved and contested by the government and people”(Kyaw, 2015, p. 50). Going further, Uddin (2020, p.4) argues that Myanmar’s dealing with the Rohingyas is not just a manifestation of their non-citizenship; it is precisely a practice meant to “reduce the Rohingyas to a status lesser than that of human beings”, and thereby push them into a ‘subhuman life’.

## From diaspora to digital diaspora: revisiting a complex transformation

Diaspora is a concept subject to various definitions and interpretations (Ponzanesi, 2020). It is defined “as a set of relationships between the homeland, which functions as a centre of gravity, and a periphery of nodes—communities, groups and individuals—who relate to the territory of origin as a centre of gravity but live in different parts of the world” (Ben-David, 2012, p. 461). Earlier studies mainly considered the dispersed population as diaspora, i.e., the Jewish, Greek and Armenian communities in exile. Today, this term shares meanings “with a larger semantic domain that includes words like an immigrant, expatriate, refugee, guest worker, exile community, overseas community, ethnic community” (Tölölyan, 1991, p. 04). While a distinction between various forms of diasporas is plausible, community belongingness, a sense of loss, nostalgia and transnationality are universal features embedded in almost all diaspora communities.

The influence of information and communication technology in the past decade has not only transformed the ways and scales of interactions among diaspora members but also led to a substantial transformation in the modern understanding of diaspora (Marat, 2015; Lobbé, 2021; Bernal, 2020). From structured networks of migrant websites to more personalised WhatsApp and Facebook groups, the wide variety of digital layers is taking the notion of diasporic organisations to a new height (Dekker et al., 2018; Dumitriu, 2012). In this changing milieu, hybrid and multifaceted migrant identities are constructed and negotiated through various discursive means (Georgalou, 2021). The advancements and proliferation of such online communication technologies encouraged a new form of virtual diasporic connections and networks that is gaining prominence as the digital diaspora. This connection reminds the members of “where their roots are, their original home, their sense of belonging, their community” (Ponzanesi, 2020, p. 983).

Emerging scholarship has started to accentuate the evolving nexus between technological advancement, the proliferation of social media and the ability of diaspora populations to create networks and become part of transnational diaspora networks (Alonso & Oiarzabal, 2010; Kapur, 2010; Alunni, 2019). In digital migration studies, research has made a significant contribution to understanding refugees’ engagement with social media and other digital tools to stay in contact with transnational families during their migration, as well as during their process of settlement in the host countries (Alencar et al., 2018; Kaufmann, 2018; Leurs & Smets, 2018). Recent studies on digital diasporas also bring together complex intersections of technology, culture, political economy and agency (Bernal, 2020). For instance, in contrast to the earlier opinion of celebrating digital media as liberating and empowering for marginalised groups (Titifanue et al., 2018), more critical analysis now raises questions regarding the outcome of digital empowerment and whether such tools can bring about changes in the political and social discourse (Taylor & Meissner, 2020; Latonero & Kift, 2018; Papacharissi, 2015).

Scholars also attempted to reveal how big corporations and states use digital platforms to extend their centralised power and use it for surveillance purposes when necessary (Bircan & Korkmaz, 2021; Zuboff, 2019). Furthermore, social media posts and activities are being systematically monitored to validate or disprove the LGBTIQ identity of many refugees requesting asylum in European countries. Targeted social media campaigns and recruitment of paid agents to monitor the Facebook activities of migrants have also become one of the strategies for governments to control and counter immigration (Andreassen, 2021; Brekke & Thorbjørnsrud, 2020). Scholars have also started to highlight the potentially pernicious role of digital tools in stimulating ‘digital nationalism’ by dividing public debate through the establishment of filter ‘bubbles’ and ‘echo chambers’ in which individuals with homogenous political thinking promote ethnocentric ideas and content that align with their views and opinions. (Mihelj & Jiménez-Martínez, 2021; Cardenal et al., 2019; Dubois & Blank, 2017).

There is also growing criticism of the dominant strand of literature on digital migration studies that are heavily focused on the Global North, particularly Europe. Such criticism has become more widespread following the so-called refugee crisis in Europe in 2015, which demands a decentralised approach to diaspora and forced migration studies and input from the perspective of the Global South (Leurs & Smets, 2018). For instance, despite the scale and extent of the Rohingya crisis in Southeast Asia, literature that offers a nuanced understanding of their digital resistance and resilience remains inadequate. To date, we have come across only a few studies that partly address the digital engagement of Rohingya refugees (e.g., Aziz, 2022; Ansar & Khaled, 2022; Abraham & Jaehn, 2020). Taking a gender lens, Ansar & Khaled (2022) presents how social media has widened the scale and scope of Rohingya women activists’ civic participation in exile. In his latest work on Rohingya digital engagements, Aziz shows how digital platforms compensate for the community’s social and spatial immobility through “digitally mediated transnational care” (Aziz, 2022, p. 01). In another recent contribution, he also presents how “the affordances of social media platforms” have facilitated Rohingyas negotiating their protracted experiences of suffering (Aziz, 2022a, p. 4082). With a mix of online and offline platforms, Abraham and Jaehn’s study (2020) shows how “diasporic Rohingya actions go beyond readily understandable demands for justice, accountability, redress” and consciously, or otherwise, take steps to reaffirm collective Rohingya identity (p. 1056). Adding onto these unfolding dynamics, this article brings an organic reflection on this ‘digital diaspora in the making’ and their forms of engagement in online platforms and its manifold implications.

## Theoretical and methodological framework

The paper’s theoretical foundations are based on the premise that scattered and oppressed ethnoreligious minorities or endangered groups, frequently organised in diasporas, use the internet to “re-create identities, share opportunities, spread their culture, influence homeland and host-land policy, or create debate about common-interest issues using electronic devices” (Alonso & Oiarzabal, 2010, p. 11). In an “unevenly interconnected world”, digital platforms provide spaces and offer alternatives to tap resources and capacity building, creating links and connectivity for dispersed communities (Ponzanesi, 2020, p. 978). This virtual space acts as “crucial protagonists” (Marino, 2015, p. 01) to manifest “diasporic identity, political activism and sentiment towards homeland” (Marat, 2015, p. 01). Besides, the “low barriers to entry and exit, and non-hierarchical and non-coercive” nature of the internet provides diasporas with a complete package of ‘benefits’ to pursue their socio-political and cultural endeavour on digital platforms (Brinkerhoff, 2009, pp. 47–48). Apart from creating a transnational network of solidarity, it allows the “expression of diverse and contested views” of the community members (Titifanue et al., 2018, p. 02).

Given the access to digital platforms by the exiled Rohingyas and the scale and extent of their virtual engagement, we have employed digital ethnography (Pink, 2013) as a method for observing their activities in virtual space. It is argued that such internet-based observations “can creatively deploy forms of engagement to look at how these sites are socially constructed and, at the same time, are social conduits” with ‘online traces’ such as retweets, hyperlinks and hashtags (Hine, 2009, p. 11). The rapidity with which people across several platforms keep up to date and their willingness to argue and voice opposing perspectives when appropriate via these interconnected networks is even more remarkable (Postill & Pink, 2012). These diverse and fast-changing characteristics have also led to more nuanced and innovative methods of using online ethnography (Pink et al., 2016). We use a ‘discourse-centred’ (Androutsopoulos, 2009) online ethnography and employ a ‘screen-based’ discourse analysis that concentrates on “systemic longitudinal and repeated observations of online-discourse” (Georgalou, 2021, p. 4).

In doing online ethnography, it is also pertinent to acknowledge the limitations of virtual platforms on the findings. For instance, Dicks et al. (2005, p.128) caution that the internet should never be read as a ‘neutral’ observation space, as it always remains a fieldwork setting and, as such, a researcher’s data selection and analyses are always biased by agendas, personal histories and social norms. Besides, the drawback of these research options is that membership of these communities is inherently restricted to the digital ‘haves’ (or at least those with digital social capital) rather than the ‘have nots’, and ethnic/gender digital divides strongly persist (Murthy, 2008). Therefore, like any other data source, social networking websites should be treated in a nuanced or layered fashion and contextualised properly (Murthy, 2008, p. 846).

Informed consent appears to be a crucial aspect of researching online communities. Whether and to what extent informed consent is required remains a contested topic (Willis, 2017, p. 3). According to Association of Internet Researchers (AoIR) ethical guidelines, public forums can be considered more public than conversations in a closed chatroom (Ess and AoIR, 2002: pp. 5, 7). Hence, ‘the greater the acknowledged publicity of the venue, the less obligation there may be to protect individual privacy, confidentiality, right to informed consent, etc.’ (Ess and AoIR, 2002, p. 5). Whiteman (2012, p. 9) also suggests it is preferable to take a contextualised approach to each online situation instead of adhering to generalised, context-free principles. Considering the above observations, the researchers sought ethical guidance from their respective institutions and received prior ethical approval before conducting their research. Furthermore, given the sensitivities of the topic, individual posts, images and tweets shared in this article are blurred to maintain confidentiality and any information that discloses the individual identity has been carefully revisited and avoided when referring to the data and images.

For data collection, we followed two major social media platforms: Facebook and Twitter. We analysed relevant Facebook and Twitter accounts and determined the top ten accounts based on the number of followers, the frequency of postings and the volume of comments. The Facebook pages and Twitter accounts were identified using eight search terms: ‘Rohingya refugee’, ‘Rohingya genocide’, ‘Rohingya women’, ‘exiled Rohingya’, ‘Rohingya activist’, ‘Arakan Rohingya’, ‘United Nations and Rohingya’ and ‘Rohingya in Bangladesh’. The qualitative corpus comprised posts and tweets that were open to the public. The study covers the period from August 2019 to August 2021. One of the authors has near-native fluency in the Rohingya language and initially attempted to explore Facebook pages and Twitter accounts on the Rohingya language despite the absence of Rohingya script, which remains an oral dialect (Aziz, 2022a, p. 4073). It did not yield significant success, prompting searching for relevant online platforms and social media tools using English.^1^ For instance, UNHCR in Malaysia has a dedicated website on “The Rohingya language”, which is written in Latin alphabets.^2^ Therefore, language and its digital representation bring another important dimension when exploring the Rohingya community’s social media engagement. The use of English in contemporary diaspora presents an ‘interesting cleavage’, as a native language is often considered a salient marker of collective identity (Kumar, 2018). We avoid the discussion at length here as it goes beyond the scope of our study; nonetheless, it is a crucial aspect to shed light on in future research on the Rohingyas. Nevertheless, we do acknowledge there are other platforms, including more private platforms like WhatsApp (Aziz, 2022a). We did not pursue these, as our purpose was to retrieve online and easily accessible data to any random visitor to those webpages.

For analysis, the transcripts of Facebook discussions and tweets were manually inserted into a dataset. This dataset was then transferred and analysed using Max Q.D.A. software to categorise the thematic contents, frequency of words, hashtags and recurring themes. Through this categorisation and coding, key themes emerged. These themes were then merged and clustered thematically, as detailed in the following section.

## From exile to online: emergence of a digital Rohingya diaspora

Multiple trajectories, including the construction of a collective Rohingya identity, political and social mobilisation and solidarity with fellow Rohingyas through providing information and long-distance emotional and material support, have emerged as the recurring features of their digital engagements. The internet has effectively bridged geographical barriers amongst Rohingyas with similar concerns by functioning as a ‘mobilising structure’ (Kumar, 2018, p. 11). The proliferation of virtual engagements creates conditions where individuals come together on shared hopes, purposes and objectives, which Tsagarousianou (2007) defined as ‘co-presence’ and Marino (2015) refers to as ‘space making’.

To detail out these manifold engagements, we conduct a two-pronged analysis of the Rohingya diaspora’s digital participation. First, we begin with a focus on the *scale of engagement*, bringing attention to their growing participation in digital space. Second, we take a more in-depth look at the *domains of engagement*, highlighting the key aspects that predominate in the interaction that takes place online.

### Scale of engagement

Owing to rapid development and relatively easier access to technologies, more Rohingyas are embracing digital platforms to interact with one another and the greater international communities. For example, only three of the ten most followed Facebook pages were created before 2017. Table 1 presents an overview of the ten most popular (in terms of membership) Rohingya Facebook groups active in different parts of the world, where membership reaches as high as 223,000 as of August 2021 (see Table 1).^3^

**Table 1:**
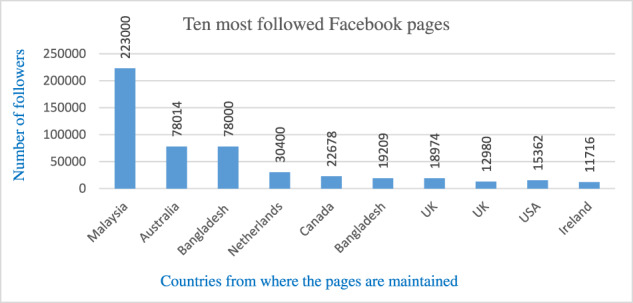
Membership pattern in ten most popular Rohingya Facebook pages.

Similarly, among the top ten Twitter accounts, only four accounts were active before 2017. A relatively recent phenomenon is the exponential increase in the number of Twitter accounts and followers of notable Rohingya activists. Table 2 illustrates the number of followers each of the top ten active Twitter accounts has. Another notable fact is that seven of the top ten account holders are based in North America and Europe (see Table 2).^4^

**Table 2:**
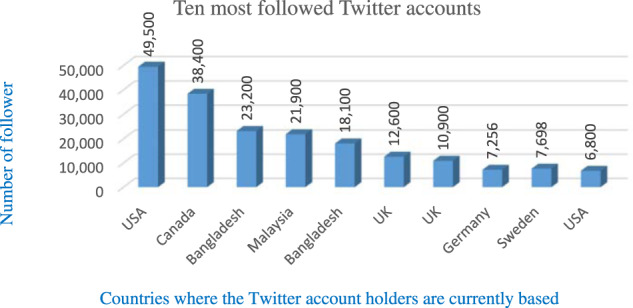
Membership pattern in ten most popular Rohingya Twitter accounts.

**Fig. 1:**
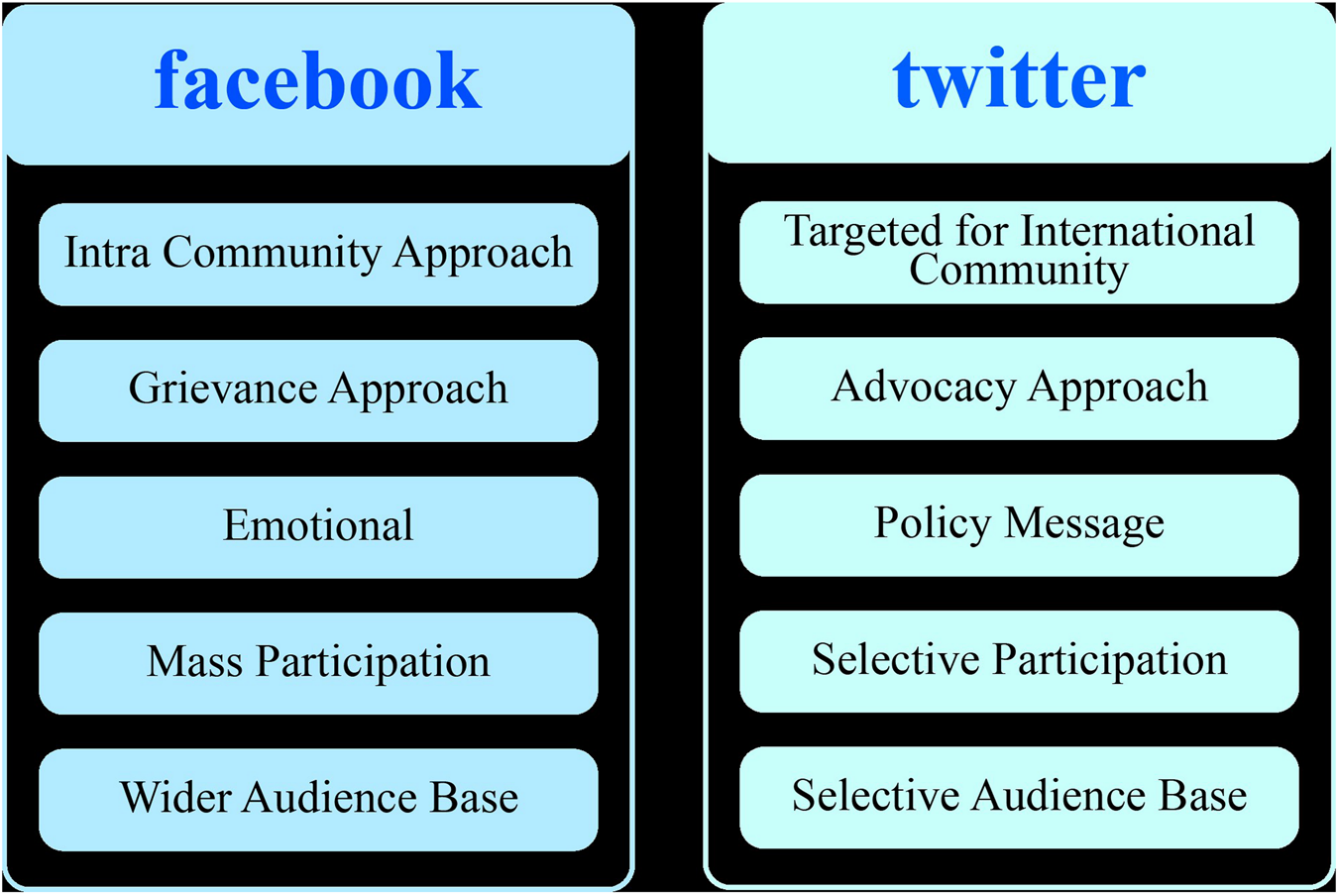
The difference in issues and characteristics between Facebook and Twitter. Rohingya diaspora on Facebook and Twitter: a comparative reflection.

A notable distinction is apparent between the two social networking platforms. As illustrated in Fig. 1, posts and discussions on Twitter were more policy-oriented and directed at international audiences and activists. On the other, Facebook discussions mostly covered emotional aspects, focusing more on nostalgia, shared grievances and experiences of escape and everyday survival in the host country. Among the Facebook pages we analysed, we found an intra-community approach in their online interactions. Twitter users frequently engage with and speak to more diverse audiences, displaying an international perspective (see Fig. 1). The geographical diversity among users is another important marker of difference within the community of users. For example, Rohingya activists in the United States (U.S.), Canada, Europe, the United Kingdom (UK) and Australia are more active on Twitter. On the other hand, Rohingyas in Bangladesh and Malaysia engage mainly on Facebook to disseminate information and engage with the broader community members in a virtual space.

Overall, three factors have contributed to Rohingya communities’ expanding activities on web platforms. First, the Rohingya crisis has garnered the global spotlight, particularly since 2017. This internationalisation has encouraged a section of the Rohingya diaspora to actively draw attention to their plight (Ansar & Khaled, 2022). Second, the proliferation of digital connectivity has offered new avenue to foster a collective Rohingya identity that is, otherwise remain suppressed in the face of long-standing marginalisation, statelessness and geographically dispersed settlement. Third, the Rohingyas’ mobility constraints and the aftermath of the pandemic further contributed to their reliance on social media platforms to voice their opinions.

### Domains of engagement

**Fig. 2:**
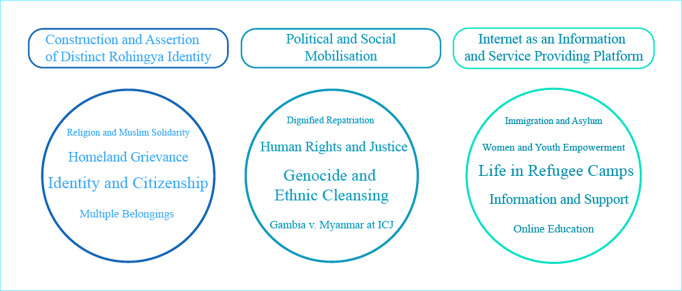
Major discussion themes on web platforms. Three major components of Rohingya diaspora’s digital engagement.

Discussions on both social media platforms cover a wide range of subjects. As illustrated in Fig. 2, a myriad of issues continues to surface on social media platforms, including Rohingya genocide and ethnic cleansing, homeland grievance, citizenship rights, current political deadlock and assertion of a distinct Rohingya identity (see Fig. 2). Furthermore, the widespread use of digital communication among the young population led to a noticeable increase in engagements with international and national humanitarian organisations.

Based on the initial coding and clustering, we identified three major components of digital engagement, as illustrated in Fig. 4: construction and assertion of distinct Rohingya identity, political and social mobilisation and use of the internet as an information and service-providing platform. The following section explains the issues in detail.

#### Claim, construction and assertion of distinct Rohingya identity

Social media platforms have created a new possibility for the exiled Rohingyas to contest the official Burmese narratives, which portray them as illegal settlers. Although the genesis of Rohingya identity has been fraught with ambiguity and hybridity, there has been a “working consensus” among the Rohingya diasporas in reinforcing their ethnic identity (Goffman, 1959). They attempt to forge this working consensus through a wide range of online performances in their everyday digital activities. Spatial nostalgia, for instance, referring to the Rakhine state as Arakan, the regional capital Sittwe as Akyab, reciting poetry on the Kaladan river and reminiscing about their relatively peaceful past under the Arakan kingdom not only connects them to their motherland, but also indicates the relevance of residual memories of their ‘lost home’. Besides, memories of violence, persecution and forced displacement, deaths of loved ones and separation from family “contribute to the bonds of community and connectedness” (Bernal, 2010. pp. 123–124) and serve as the ‘catalysts’ to form a collective identity (Alonso and Oiarzabal, 2010).

**Fig. 3:**
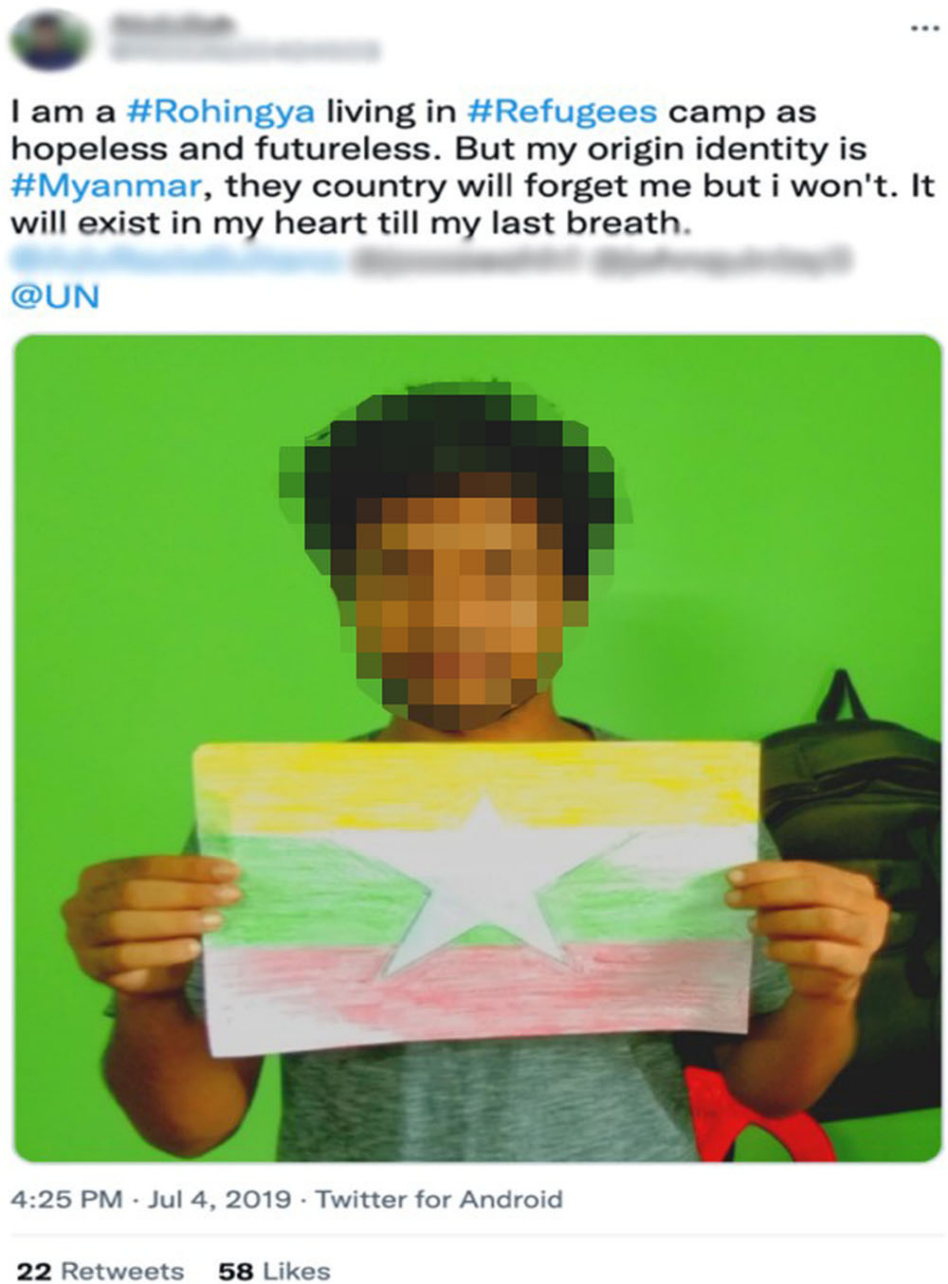
A Rohingya refugee holding Myanmar’s flag to assert his Burmese identity. Rohingya youth asserting multiple belongings on Twitter.

This identity construction and cultural reproduction are manifested in organised content and narratives illustrated on social media. They frequently present historical evidence, figures and visuals to support their claims of Rohingya ancestry in Arakan, which contradict the official narratives of the Myanmar government and ultra-nationalist Buddhist political and religious organisations. This resolute defending of ethnic Rohingya identity, referring to Rakhine state as their homeland and claiming right to return and citizenship of Myanmar, function as the ‘centre of gravity’ for users and the way they engage on virtual platforms within and beyond the community (Ben-David, 2012). Nevertheless, many Rohingya also strongly affiliate with their roots in Myanmar and assert their ‘Burmeseness’, thereby embracing multiple identities (see Fig. 3). As Fig. 3 shows, a young Rohingya who considers Myanmar an integral part of his identity not only reveals the fluidity of identity formation, but such expression of a multi-layered identity also confronts those who embrace a more ethnicised and religious identity.

**Fig. 4:**
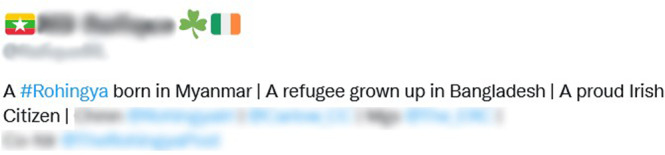
An Irish Rohingya human rights activist claiming multiple identities. Rohingya diapsora activist claiming multiple identities on Twitter.

Such hybridity of Rohingyas’ online identity derives from the flexibility and temporality that one can assert on social media given the unfixed nature of cyberspace—‘nothing is real’ (Marat, 2015, p. 9). This exercise of asserting a collective identity involves a process of constant adjustment and readjustment predicated on the time, space and social context of the host countries. For example, Fig. 4 shows how the digital activities of many Rohingya diaspora members reveal their ability to preserve local, transnational and global feelings of belonging simultaneously. The illustration of this Twitter activist’s profile reveals how, depending on the situation, their online advocacy and dynamic interactions of multi-layered belonging can represent both pluralistic and localised perspectives, switching between the two, if and when necessary (see Fig. 4). It also demonstrates their capacity to maintain traditional markers of ethnic, religious and cultural identity while also claiming different forms of membership, and thereby functioning as cultural intermediaries. Such flexibility puts them in a strategic position that allows them to support the issues of the Rohingya community, transcend ethnic boundaries and become international human rights activists. This multi-layered and elastic identity construction also indicates that key parts of the dynamics of digital engagement include staying flexible and being ready to keep moving beyond the ‘peripheral ties’ between diaspora and non-diaspora actors (Ben-David, 2012).

**Fig. 5:**
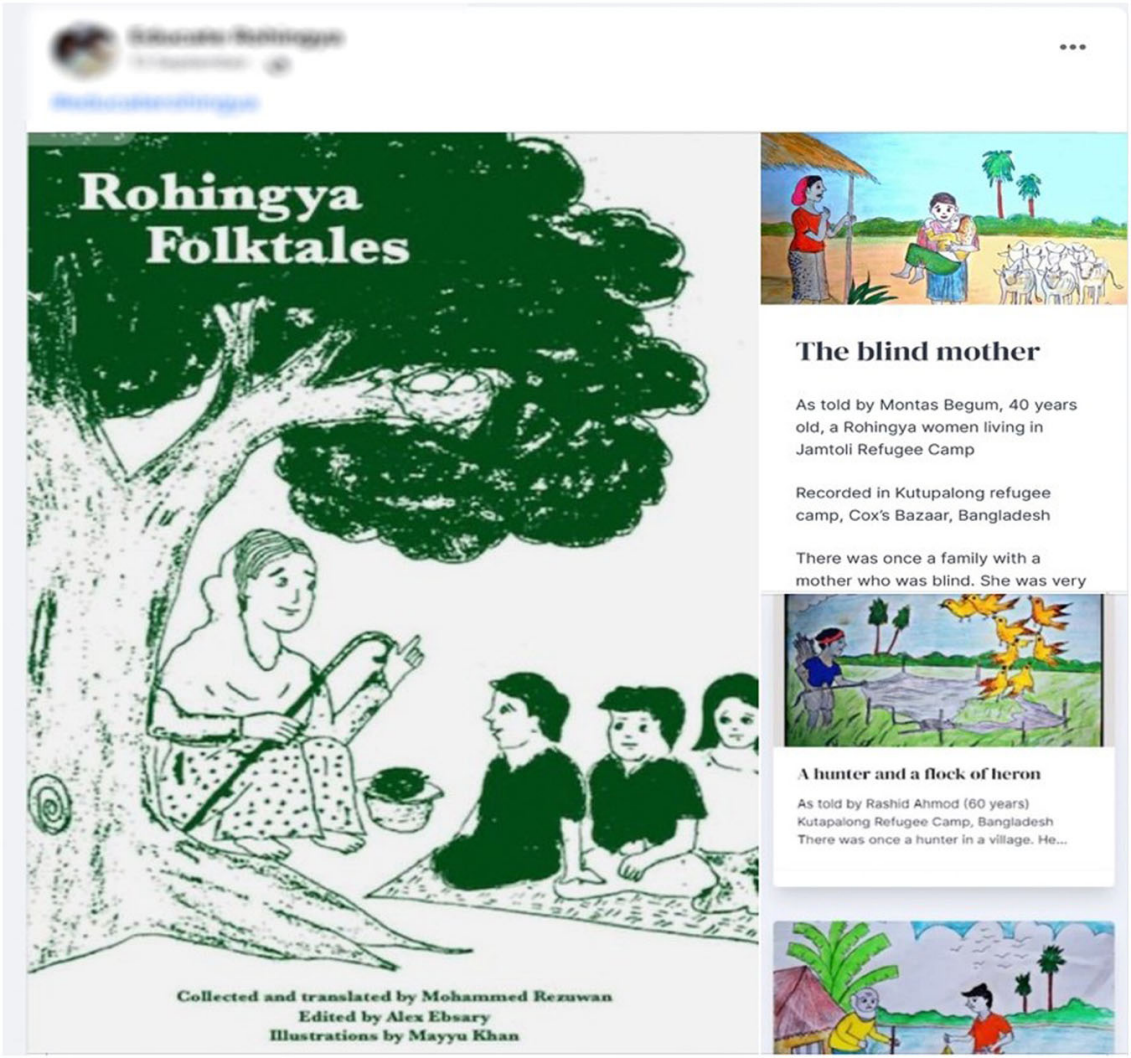
Online collection of translated Rohingya folktales. Dissemination of Rohingya folklore online via Facebook.

In addition, iconic images, traditional cuisines and other symbolic markers continue to appear online to foster cross-border feelings of collective identity and forge consensus. Music and folklore in the Rohingya language are frequently shared on social media platforms (see Fig. 5). Despite their scattered settlements, young Rohingyas can maintain ties with their culture and heritage by participating in online classes taught in their native language. Social media platforms often host cultural and art competitions to revive and promote Rohingya art and culture, which is vital to preserving and promoting their ethnic identity and tradition.

As part of this identity-building effort, social media posts and tweets promote particular hashtags. Hashtags are not just a facet of online culture; they are also a means of expression that has helped users create a “hashtag sociality” that grows out of the interconnections and relevant threads (Postill and Pink, 2012, p. 9). The widespread use of hashtags has helped internationalise the Rohingya’s situation while assisting with identity development, self-image building and social and political mobilisation. Using hashtags such as #**Rohingyarefugees, #Rohingya, #Rohingyaremembranceday** and the like, Rohingya diasporas have created a network of interactions with fellow community members and beyond. The hashtags also assist them in locating each other, strengthening the notions of solidarity and identity assertion of what Boyd (2010) refers to as “networked publics” of imagined communities. This identity-building gradually becomes identity politics as the online users deploy their identity to shape the perception of belonging (Kumar, 2018).

Other than claiming and affirming Rohingya identity, there are efforts to revive other aspects of their identity, such as religion. For example, Rohingya Vision, the first Rohingya satellite news station with more than 240,000 Facebook followers, begins its live news with an invocation to Allah and the Prophet Mohammed. Political updates are posted regularly about the hardships Muslim communities face worldwide. Such display of religious rituals may obscure the boundaries between religion and ethnicity in constructing the Rohingya identity. Although it serves the purpose of creating a sense of solidarity and connecting with the global Muslim identity, such an exhibition may “engenders a cathexis between a community of believers and a people joined by suffering” (Abraham and Jaehn, 2020, p. 1058).

#### Political and social mobilisation

Going beyond framing and asserting collective Rohingya identity, social media access also facilitated a new political and social mobilisation channel. Rohingyas employ a unified, coherent, human rights-based discourse on digital platforms to articulate their political grievances and present a coordinated call for action. Rohingya Campaigners, particularly on social media sites such as Twitter, commonly utilise hashtags to spread the news of ongoing events and frequently tag local and international organisations and known global human rights campaigners. For instance, there has been an organised social media campaign during the Gambia vs Myanmar case at the International Court of Justice (ICJ) on Twitter and Facebook. Numerous hashtags such as **#StopRohingyaGenocide**, **#CallitaGenocide and #JusticeforRohingya** dominated the social media platforms during the ICJ hearing in the Netherlands in December 2019. Participants frequently publish images of torture, accounts of state-sponsored abuse against women and amateur news clips from the Rakhine State, including footage of burning mosques, villages and dead bodies following military crackdowns.

**Fig. 6:**
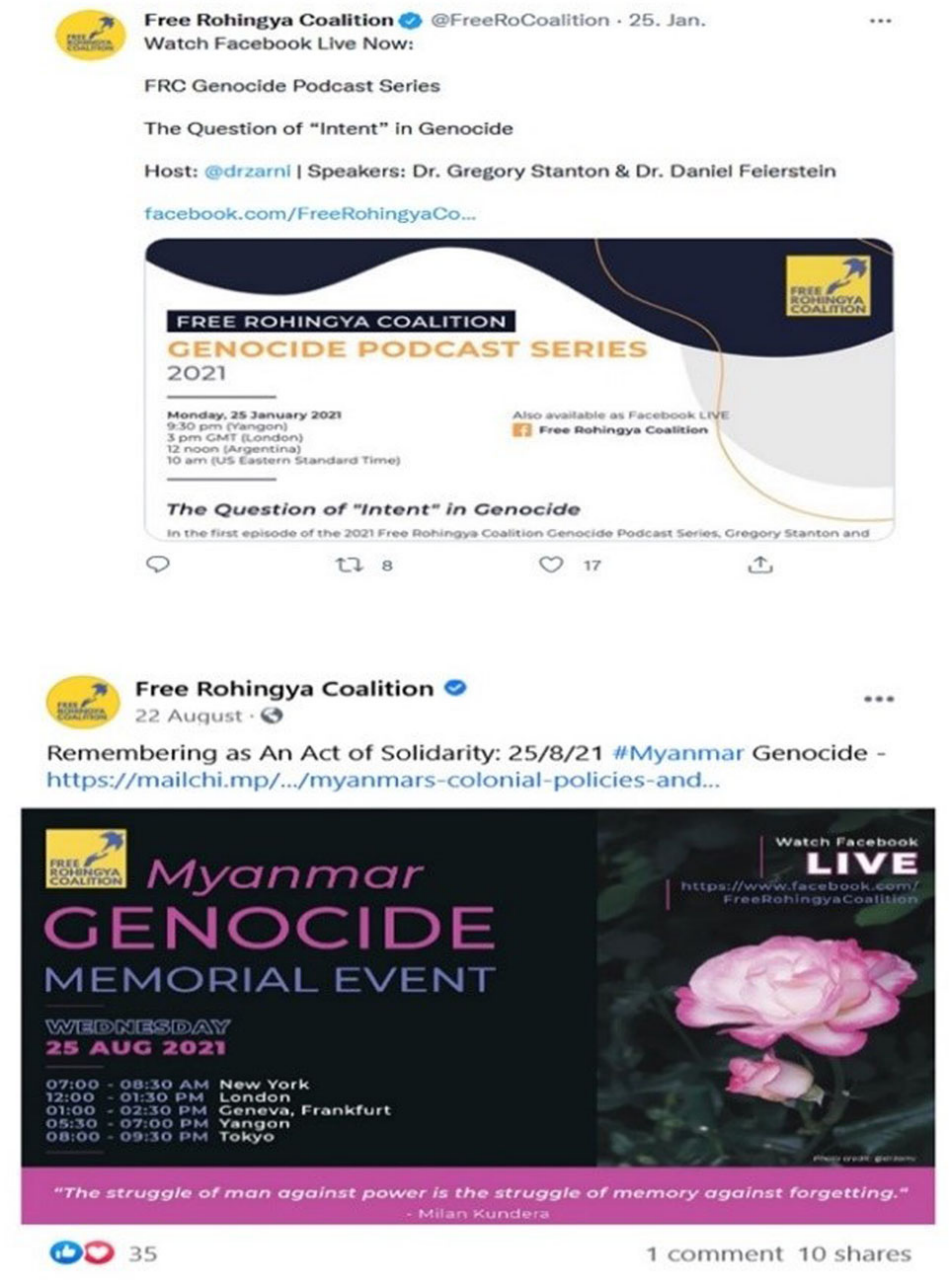
Online seminars are used to internationalise the Rohingya plight. Rohingya diaspora platforms hosting webinars and podcasts on genocide recognition.

Numerous webinars and online conferences have been organised in recent years to commemorate the Rohingya genocide in Myanmar. Figure 6 illustrates that Facebook pages and Twitter account covering these events constantly provide updates, disseminate important messages and even live stream the events. Such online mobilisation has taken the lead in political lobbying, awareness-raising and updating the international community about their shared struggle in Myanmar and in exile (see Fig. 6). Through this categorical display of suffering and dissemination of information, diaspora members function as the transporters of cultural and political views (Clifford, 1992). Thus, online platforms have become a crucial ‘mobilising structure’ and ‘focal hub’ to pursue their political goals (Kumar, 2018, p. 11).

This coordinated web activism offers the diaspora members a “connective opportunity structure” in agenda-framing and in pursuing a concerted effort to pin their claims and concerns to the broader international system (Kumar, 2018, p. 11–12; Marat, 2015, pp. 6–7). By strategically using digital platforms, they seek wider recognition and support from the global community and mobilise opinions in their favour. Through real-time engagement with state and non-state actors, they continue to advance their social and political objectives. Since the February 2021 military coup, such web-based activism has started to connect with the broader Burmese civil resistance against the rule of the military. Human rights advocacy and the cultivation of an “oppositional consciousness” have helped them draw attention to the complex political situation in Myanmar and transmit information about the decades-long injustice they have suffered. Framing a human rights discourse and developing an ‘oppositional consciousness’, they spotlight complex political unfolding in Myanmar and disseminate information about how injustice has been done to them for decades (Mansbridge, 2001). This culture of resistance and oppositional consciousness help establish them as an emerging political voice beyond the community boundary.

**Fig. 7:**
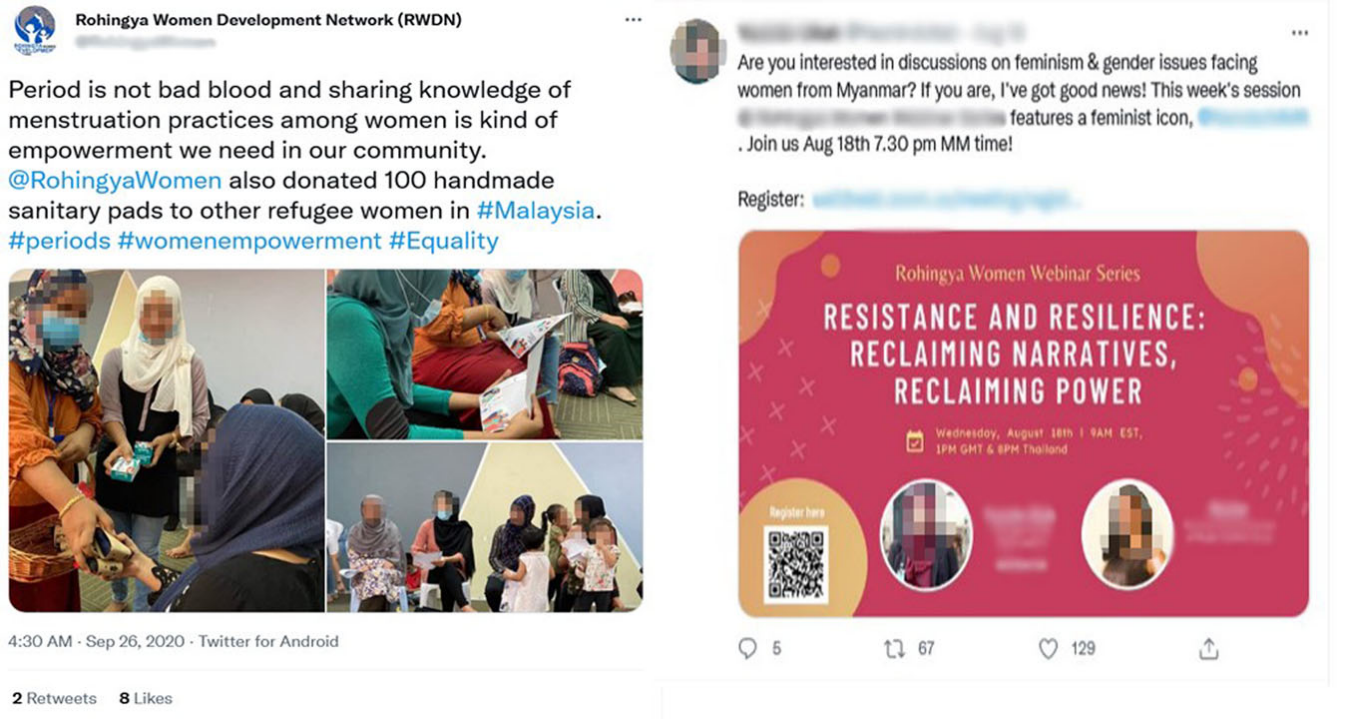
Rohingya women bring gender issues to the forefront of their struggle. Rohingya women activists raising gender awareness on social media.

A significant development amid such evolving political and social activism is the active participation of women and young people. For example, Fig. 7 shows how Rohingya women are taking a prominent role in shaping the political and social narratives on their displacement (see Fig. 7). They continue to challenge societal and patriarchal norms as they shed light on gender-sensitive issues, including menstruation hygiene, birth control and adolescent girls’ health, as a recent study shows (Ansar and Khaled, 2022). This is especially evident among the younger generation, many of whom fled Myanmar as children and have been profoundly influenced by their ‘long-term exposure to exiled life’ (Titifaune et al., 2018). Such active participation not only shapes their understanding of Myanmar but also adds an innovative perspective to pursue their collective gendered struggle. Digital platforms, therefore, contribute to the connectedness and bonds with their root and create an opportunity to coordinate lobbying and cultural brokerage by linking, managing and collaborating with relevant stakeholders (Andén-Papadopoulos, Pantti (2013), p. 2188).

#### Social media as an informational and service-providing platform

**Fig. 8:**
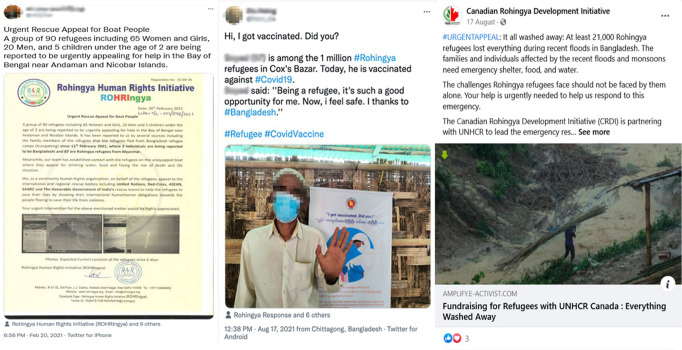
Social networking sites have emerged as a vital source of information and community services. Rohingya activists leveraging social media to disseminate relevant information.

There is a broader consensus among scholars on how digital technologies have become an essential means to maintaining transnational connectivity, information and caregiving practices (Kaufmann, 2018; Leurs & Smets, 2018). This connectivity helps diaspora members to remain involved in everyday experiences and to fulfil their familial, social and communal responsibilities. For many Rohingyas, online communities serve as the initial point of contact for various services. Increasingly, digital platforms foster a sense of collective responsibility among social media users. Figure 8 shows, how social media users often function as first responders to disseminate emergency information, such as news about Rohingyas being stranded at sea, emergency blood donation and urgent response to flood victims in Bangladesh refugee camps. They also coordinate fundraising and encourage hesitant community members to get vaccines, against Covid-19 for example. Several petitions have been initiated online to protest what the Rohingya people see as unfavourable government policies, such as building barbed-wire fences around refugee camps (see Fig. 8). This networked, transnational solidarity that is shaped by the daily experiences of immobility is something Aziz refers to as “mediated care practices beyond nation-states and borders” (2022a, p. 14).

Online community members regularly advise each other on immigration-related bureaucratic procedures, Covid-19 regulations and health and refugee-rights related services. They post regular updates on the political situation in host countries and on policy changes that might impact their current status in the respective countries. In doing so, they also transcend the public-private boundaries of everyday life, as they create a direct communication channel between social media users. This virtual network of emotional and informational support systems and the ‘home feeling’ is what Marino refers to as a “community of comfort” (Marino, 2015, p. 2).

The creative use of digital technologies also turned the online platform into a source for distance education. Users frequently post information on scholarships for refugees and offer online English language courses and skills training. It became particularly prevalent during the Covid-19 pandemic as a viable alternative to classroom instructions. For young Rohingyas in refugee camps in Bangladesh, access to online education provides a welcome opportunity to expand their horizons beyond the Burmese curriculum. Many young refugees pursue English language learning, as it creates opportunities to work with local and international NGOs and third-country resettlements. As Díaz Andrade and Doolin (2016) demonstrate, this online community provides diaspora members with five distinct affordances to help them adapt to their new home’s social and cultural challenges: “to participate in an information society, to communicate effectively, to understand a new society, to be socially connected, and to express a cultural identity” (p. 405). Arguably, a certain level of trust is involved in such virtual exchanges, which may derive from users’ shared experiences as persecuted refugees. Thus, digital platforms generate a sense of ‘togetherness’ and ‘belonging’ since they give a virtual social space, transmit critical information and motivate each other about new life prospects and opportunities (Ponzanesi, 2020; Alunni, 2019).

## Embodying exile through collective identity, solidarity and civic mobilisation

The empirical discussion above shows that for Rohingya diasporas in exile, personal experiences of persecution, migration, statelessness and resistance are important experiences for forming identity, solidarity and political engagement. These lived experiences of diaspora constantly remind Rohingyas of their otherised identities as refugees, stateless, asylum seekers, immigrants, Muslims and Asians. Engaging in digital platforms has thus become a means of (re)creating and (re)affirming one’s identity and sense of belonging in response to otherisation and exclusion from mainstream communities. Furthermore, the myriad of digital engagements manifested on social media platforms has redefined the notion of connectivity, territoriality and civic activism among displaced Rohingyas, where, through self-representation, they attempt to claim their space by contesting and reshaping narratives that govern their everyday lives.

In digital platforms, there is a collective quest to promote a distinct Rohingya identity, which we have framed as the ‘pursuit of Rohingyaness’. The defining characteristic of Rohingyaness is the vigorous defence of a distinct Rohingya ethnicity that is deeply rooted in Myanmar’s social and political landscape. In their quest for Rohingyaness, they regard themselves as “bridge-builders” (Müller-Funk, 2020, p. 1120), connecting not only the members distributed across diverse spaces but also with the larger international community. This portrayal of collective memories of suffering and feelings of victimhood resonates with the idea of “platformised pain” (Chouliaraki, 2021, p. 10). Such platformised pain conveyed through virtual affinity likely contributes to offline interactions and community development, resulting in a sense of belonging to a broader diasporic network (Marino, 2015, p. 06). In doing so, they craft “affective fabrics”, while reviving a sense of belonging and solidarity based on a shared history and homeland roots in cyberspace, which they “inhabit at the time of the permanently ephemeral” (Tsaliki, 2003, p. 174). Given that most Rohingya “will never know most of their fellow members, meet them, or even hear of them” in offline space, the pursuit of Rohingyaness in cyberspace through the dissemination of memories, culture, symbols, and myths is analogous to the imagined community (Anderson, 2006, p. 49). Thus, a parallel can be drawn between Benedict Anderson’s concept of an imagined community becoming a nation and how modern diasporic relationships are envisioned as transcending borders through these digital platforms. As a result, cyberspace has enabled them to connect with fellow Rohingyas in the diaspora and “homeland”, strengthening their feeling of shared imagined Rohingya Community and diasporic consciousness.

In addition, the Rohingya diasporic identity is not static, but rather shaped by a complex set of interwoven diasporic experiences. This transformation has been shaped by the socio-political attributes of the host country and overlapping intersectional factors such as age, gender, education and geographic location, among others. These multiplicities and fluidities of identity are something that Marat (2015) refers to as becoming (ongoing process) a mixture of endogenous and exogenous identity. In this complex process of self-representation and identity-making, social media users routinely transgress their symbolic ethnic and cultural boundaries by engaging with wider global audience who supports their struggle. Doing so, they facilitate political dissent and resistance against hegemonic Burmese military regime, both within and beyond their community, which Ansar and Khaled (2022, p. 291) terms as ‘conditional solidarity’—exhibited through Rohingyas’ growing yet muted collaboration with other Burmese ethnic groups following the military coup in Myanmar in 2021.

Such political mobilisation, which has become an essential marker of the Rohingyas’ cyberspace engagement, is dependent on a wide variety of factors such as spatiality, positionality, the availability of cyber capital and the host country’s political opportunity structure, among others (Kopchick., et al., 2021; Bernal, 2018; Graham & Khosravi, 2010). For the geographically scattered Rohingya, considering their immobilities and offline constraints, social media has emerged as a “virtual public-square” (Reyaz, 2020, p. 23) that not only fosters everyday connections but also provides a crucial space for social and political mobilisation. Furthermore, their shared pain, loss and experience of displacement resonates with their urge for self-representation, reclamation of political identities and expression of diasporic agencies. While digital platforms have been the most deterritorialised and globalised space, most Rohingya digital engagements centre on the Rohingyas themselves, as they seek to reterritorialise online political spaces by injecting their narratives and establishing political co-presence (Graham & Khosravi, 2010). In the form of ‘leverage politics’, these online mobilisations, with coherent political narratives, facilitate transnational lobbying and agenda setting and influence public opinion and decision-making in their favour (Keck and Sikkink, 1999, p. 72). With this organised civic activism on virtual platforms, the Rohingya community members manifest “their collective sense of self, who they are and what they stand for” (Gerbaudo and Treré (2015), p. 865). The practices of concerted civic participation that they cultivate in online forums strengthen not only their sense of rights, justice and community but also unearth injustices committed against them. Therefore, social media platforms have enabled the Rohingyas to mend “ruptures in the social body” and streamline the political and social arguments in their favour through virtual interaction within and beyond their community (Bernal, 2010, p.124).

## Spatiality and access: reflection on the digital divide

While social media activism has made it possible for more people to be heard online, researchers are beginning to draw attention to the disparities in diaspora participation in online communities (Schradie, 2018; Bennett and Segerberg, 2012). This brings us to yet another crucial debate—the digital divide. Understanding the digital divide requires considering the different contexts in which it might occur. Each context has its own power dynamics, and differentiating attributes can facilitate or stifle digital interactions. In addition, a digital divide does not merely denote a chain of causality in which lack of access to digital communication tools and the internet limits online civic involvement. Instead, the relevance of age, gender, education, host country environment and language, among other factors, are essential drivers in understanding the digital divide among diasporas scattered across heterogeneous spatiality.

As Norris (2001) argued, the issue of the digital divide can be broken down into three distinct, but interconnected tiers of analysis: the macro-level (economic and technological factors determine Internet access and distribution), the meso-level (political institution and related opportunities), and the micro-level (personal capacity, interest and drive as determinants of online civic participation). While analysing the digital activities of Rohingya diasporas, we found that Norris’s levels of analysis had the highest resonance. To provide more context, seven of the top ten most followed Twitter accounts are run by Rohingya activists based in the Global North. Similarly, eight out of the ten most followed Facebook pages are running from the Global North. In addition, the webinars we followed are exclusively organised by the Rohingya diaspora platforms based in countries such as Germany, the U.K., Canada, the US and Australia. There are, thus, noticeable differences in the levels of participation, capabilities and access to resources in various forms of digital activity across different geographical configurations. On the other hand, in both Malaysia and Bangladesh, in addition to the logistical constraints, refugees face an array of challenges to freedom of expression on digital platforms. This forces many Rohingya activists to remain less assertive, even as the host country’s policies have detrimental consequences on the refugees living there.^5^ Yet, it is important to reiterate the limitations of using English-only search terms, which is perhaps another factor as to why the most prominent accounts we identified were primarily based in countries where English is the first language. Therefore, we do acknowledge that there could be other accounts with more prominence in different languages that did not appear in our research.

Another noteworthy distinction between the Rohingyas in the Global South and the Global North is their settlement pattern. While the Rohingya diaspora communities in the Global North are primarily based in urban metropolises, Rohingyas in the Global South, such as in Bangladesh, Malaysia and Thailand, are primarily settled in the remote borderland, informal settlements and isolated refugee camps with limited digital infrastructure. Inequalities in civil liberties and lack of rights, discrepancies in digital infrastructure and accessibility and variances in the socioeconomic context of the host country are all likely to affect the diasporas’ capacity, interest and motivation for diasporic engagement in cyberspace (Nessi & Bailey, 2019; Norris, 2001). Thus, the comparison between different countries also illustrates how, to a large extent, digital platforms compensate for the social and spatial hierarchies within the community members. Nevertheless, the scale and scope of such connectedness are conditioned by different intersectional factors, such as access to technology, age, gender, the temporality of membership and other relevant skills and privileges.

## Conclusion

This article offers a rudimentary reflection on how Rohingyas in the diaspora engage in social media platforms and how the digital realm has contributed to forming a nascent digital diaspora. In the absence of a reference to a physical homeland and the everyday immobility paradigm they are entangled with, these platforms offer a transformative space to the Rohingyas for self-expression, civic engagements and ethnic reinforcements. Acting as a comfort zone, digital spaces not only provide material and emotional support but also enhance their self-esteem and self-awareness as part of the diaspora. Focusing on their multi-layered identity-building in cyberspace, this paper shows how these engagements constitute a space for togetherness where diasporic experiences and Rohingya political identities are constructed, contested and mediated. They constellate political arguments to engage with wider audiences who are supportive of their protracted struggle in an effort to garner international solidarity. Linking Rohingya identity within the broader socio-political spectrum of Myanmar, particularly of the Rakhine state, they directly oppose the Myanmar government’s persistent policies of referring to them as foreigners and illegal settlers.

Finally, despite social media’s potential to offer a new avenue for social enquiry, we restate its limitations. To ensure an inclusive diaspora representation, it is imperative to consider the pre-existing social hierarchies and asymmetries in a nuanced way. Therefore, future research incorporating both online and offline strategies would be a significant step forward in researching and further understanding cyber ethnographies on dispersed communities across the globe, such as the Rohingyas. Such hybrid methodological conceit, with an additional focus on key geographical locations of Rohingya diaspora settlement, would set an important precedent for future research and add crucial nodes to further unfold their transnational identity.

## Data Availability

The data that support the findings of this study are available on request from the corresponding authors upon reasonable request.

## Footnotes

1 Rohingya language is a spoken dialect almost identical to the local dialect of the Chittagong region of Bangladesh. There is an ongoing effort to incorporate it in the planned upgrade to the Unicode Standard, the global coding system that turns written script into digital characters and numbers. See Abraham & Jaehn (2020) for more details.

2 For details, please see the following website: https://refugeemalaysia.org last accessed January 08, 2023.

3 The number of memberships does not indicate the figure of Rohingyas living in the above-listed countries.

4 The country names indicate the geographic locations where the account holders are currently based.

5 For Malaysia, see https://www.arabnews.com/node/1673766/world (accessed September 30 2022). For Bangladesh, see https://www.thedailystar.net/news/bangladesh/rights/news/digital-security-act-threatens-press-freedomneeds-reformation-edit (accessed September 30 2022).

## References

[CR1] Abraham I, Jaehn M (2020). ‘Immanent nation: the Rohingya quest for international recognition’. Nations Natl.

[CR2] Alam J (2018). The Rohingya of Myanmar: theoretical significance of the minority status. Asian Ethn.

[CR3] Alencar A, Kondova K, Ribbens W (2018). The smartphone as a lifeline: an exploration of refugees’ use of mobile communication technologies during their flight. Media Cult Soc.

[CR4] Alonso A, Oiarzabal P (2010) The immigrant worlds’ digital harbors: an introduction. In: Alonso A, Oiarzabal PJ (eds.) Diasporas in the new media age: identity, politics, and community. University of Nevada Press, Nevada

[CR5] Alunni A (2019). Long-distance nationalism and belonging in the Libyan diaspora (1969–2011). Br J Middle East Stud.

[CR6] Andén-Papadopoulos K, Pantti M (2013). The media work of Syrian diaspora activists: brokering between the protest and mainstream media. Int J Commun.

[CR7] Anderson B (2006). Imagined communities: reflections on the origin and spread of nationalism.

[CR8] Andreassen R (2021) Social media surveillance, LGBTQ refugees and asylum. How migration authorities use social media profiles to determine refugees as ‘genuine’ or ‘fraudulent’. First Monday, 6(1), 10.5210/fm.v26i1.10653

[CR9] Androutsopoulos J (2009). Potentials and limitations of discourse-centred online ethnography.. Language@Internet.

[CR10] Ansar A (2020). The unfolding of belonging, exclusion and exile: a reflection on the history of Rohingya refugee crisis in southeast Asia.. J Muslim Minor Aff.

[CR11] Ansar A, Khaled AF (2021). From solidarity to resistance: host communities’ evolving response to the Rohingya refugees in Bangladesh. J Int Humanit Action.

[CR12] Ansar A, Khaled AFM (2022). Claiming space and contesting gendered refugeehood in exile: issues and factors of Rohingya refugee women’s civic engagement in diaspora. Int Q Asian Studi.

[CR13] AoIR (2002) Ethical decision-making and Internet research: Recommendations from the aoir ethics working committee. Retrieved from AoIR 2022: http://aoir.org/reports/ethics.pdf

[CR14] Arendt H (2017) We refugees. In: H. Lambert H (ed.), International refugee law. 10.4324/9781315092478). Routledge, London. pp. 3–12

[CR15] Aziz A (2022). Affective networked space: polymedia affordances and transnational digital communication among the Rohingya diaspora. Int J Commun.

[CR16] Aziz A (2022) Power geometries of mediated care: (re)mapping transnational families and immobility of the Rohingya diaspora in a digital age. Media Cult Soc 10.1177/01634437211065690

[CR17] Ben-David A (2012). The Palestinian diaspora on the web: between de-territorialisation and re-territorialisation. Soc Sci Inf.

[CR18] Bennett W, Segerberg A (2012). The logic of connective action digital media and the personalization of contentious politics. Inf Commun Soc.

[CR19] Bernal V (2018). Digital media, territory, and diaspora: the shapeshifting spaces of Eritrean politics. J Afr Cult Stud.

[CR20] Bernal V (2020). African digital diasporas: technologies, tactics, and trends. Afr Diaspora.

[CR21] Bernal V (2010) Nationalist networks: the Eritrean diaspora online. In: Alonso A, Oiarzabal P (eds). Diasporas in the new media age: identity, politics, and community. Nevada Press, Nevada

[CR22] Bircan T, Korkmaz EE (2021). Big data for whose sake? Governing migration through artificial intelligence. Humanit Soc Sci Commun.

[CR23] Boyd D (2010). Social network sites as networked publics: affordances, dynamics, and implications.

[CR24] Brekke J-P, Thorbjørnsrud K (2020). Communicating borders—Governments deterring asylum seekers through social media campaigns. Migr Stud.

[CR25] Brinkerhoff MJ (2009). Digital diasporas: identity and transnational engagement.

[CR26] Cardenal A, Aguilar-Paredes C, Galais C, Pére M (2019). Digital technologies and selective exposure: how choice and filter bubbles shape news media exposure. Int J Press/Polit.

[CR27] Cheesman N (2017). How in Myanmar “National Races” came to surpass citizenship and exclude Rohingya. J Contemp Asia.

[CR28] Chouliaraki L (2021). Victimhood: the affective politics of vulnerability. Eur J Cult Stud.

[CR29] Clifford J (1992) Traveling cultures. In: Grossberg L, Nelson C, Treichler T (eds). Cultural studies. Routledge, New York. pp. 96–116

[CR30] Dekker R, Engbersen G, Klaver J, Vonk H (2018). Smart refugees: how Syrian asylum migrants use social media information in migration decision-making. Social Media+Soc.

[CR31] Díaz Andrade A, Doolin B (2016). Information and communication technology and the social inclusion of refugees. MIS Q.

[CR32] Dicks B, Mason B, Coffey A, Atkinson P (2005). Qualitative research and hypermedia ethnography for the digital age.

[CR33] Dubois E, Blank G (2017). The echo chamber is overstated: the moderating effect of political interest and diverse media. Inf Commun Soc.

[CR34] Dumitriu D-L (2012). E-diasporas Atlas. Exploration and cartography of diasporas in the digital network. Revista Română de Comunicare şi Relaţii Publice.

[CR35] Georgalou M (2021). New Greek migrant (dis)identifications in social media: evidence from a discourse-centred online ethnographic study. Humanit Soc Sci Commun.

[CR36] Gerbaudo P, Treré E (2015). In search of the ‘we’ of social media activism: introduction to the special issue on social media and protest identities. Inf Commun Soc.

[CR37] Goffman E (1959) The presentation of self in everyday life. Garden City, New York

[CR38] Graham M, Khosravi S (2010). Reordering public and private in iranian cyberspace: identity, politics, and mobilization. Identities.

[CR39] Hine C (2009) Question one: how can qualitative internet researchers define the boundaries of their projects? In: Markham A, Baym N (eds.) Internet inquiry: conversations about method. Sage, London

[CR40] Hoffstaedter G (2019). Arrested refugee mobilities. Sojourn: J Soc Issue Southeast Asia.

[CR41] Holliday I (2014) Addressing Myanmar’s citizenship crisis. J Contemp Asia 44(3):404–421. 10.1080/00472336.2013.877957

[CR42] Ibrahim A (2018) The Rohingyas: inside Myanmar’s genocide. C. Hurst & Co. (Publishers) Ltd, London

[CR43] Jernigan W (2019). Invisible immobilities: Statelessness in Southeast Asia. Retrieved June.

[CR44] Kapur D (2010). Diaspora, development, and democracy: the domestic impact of international migration from India.

[CR45] Kaufmann K (2018). Navigating a new life: Syrian refugees and their smartphones in Vienna. Inf Commun Soc.

[CR46] Keck ME, Sikkink K (1999). Transnational advocacy networks in international and regional politics. Int Soc Sci J.

[CR47] Khaled AFM (2021). Do no harm in refugee humanitarian aid: the case of the Rohingya humanitarian response. J Int Humanit Action.

[CR48] Kopchick C, Cunningham KG, Jenne E, Saideman S (2021). Emerging diasporas: exploring mobilisation outside the homeland. J Peace Res.

[CR49] Kumar P (2018). Rerouting the narrative: mapping the online identity politics of the tamil and Palestinian diaspora. Social Media + Soc.

[CR50] Kyaw N (2015). Alienation, discrimination, and securitisation: legal personhood and cultural personhood of muslims in Myanmar. Rev Faith Int Aff.

[CR51] Latonero M, Kift P (2018). On digital passages and borders: refugees and the new infrastructure for movement and control. Soc Media + Society.

[CR52] Leider J (2018) Rohingya: The history of a Muslim identity in Myanmar. In Oxford research encyclopedia of Asian history. 10.1093/acrefore/9780190277727.013.115

[CR53] Leurs K, Smets K (2018). Five questions for digital migration studies: learning from digital connectivity and forced migration In(to) Europe. Social Media + Soc.

[CR54] Lobbé Q (2021). Multi-scale methods for reconstructing collective shapes of digital diasporas. Humanit Soc Sci Commun.

[CR55] Mansbridge J (2001) The making of oppositional consciousness. In: Mansbridge J, Morris A (eds.). Oppositional consciousness: the subjective roots of social protest. Chicago University Press, Chicago, IL. pp. 1–19

[CR56] Marat A (2015). Uyghur digital diaspora in Kyrgyzstan. Diaspora Stud.

[CR57] Marino S (2015). Making space, making place: digital togetherness and the redefinition of migrant identities online. Social Media + Soc.

[CR58] Mihelj S, Jiménez-Martínez C (2021). Digital nationalism: understanding the role of digital media in the rise of ‘new’ nationalism. Nations Natl.

[CR59] Müller-Funk L (2020). Fluid identities, diaspora youth activists and the (Post-)Arab Spring: how narratives of belonging can change over time. J Ethnic Migr Stud.

[CR60] Murthy D (2008). Digital ethnography: an examination of the use of new technologies for social research. Sociology.

[CR61] Nessi L, Bailey O (2019) Racial and class distinctions online the case of the Mexican European Diaspora on social networking sites. In: Retis J, Tsagarousianou R (eds). The Handbook of Diasporas, Media, and Culture. John Wiley & Sons, Hoboken

[CR62] Norris P (2001). Digital divide: civic engagement, information poverty, and the Internet worldwide.

[CR63] Papacharissi Z (2015) Affective publics: sentiment. technology, and politics. Oxford University Press, New York

[CR64] Pink S (2013). Doing visual ethnography.

[CR65] Pink S, Horst H, Postill J, Hjorth L, Tacchi J (2016). Digital ethnography: principles and practice.

[CR66] Ponzanesi S (2020). Digital Diasporas: postcoloniality, media and affect. Int J Postcolonial Stud.

[CR67] Postill J, Pink S (2012). Social media ethnography: the digital researcher in a messy web. Media Int Aust.

[CR68] Reyaz M (2020). Cyberspace in the post-soviet states: assessing the role of new media in central Asia. Jadavpur J Int Relat.

[CR69] Schradie J (2018). The digital activism gap: how class and costs shape online collective action. Soc Probl.

[CR70] Sengupta S (2020) Being stateless and surviving: the Rohingyas in camps of Bangladesh. In: Chowdhory N, Mohanty B (eds.) Citizenship, nationalism and refugeehood of Rohingyas in Southern Asia. Springer Nature, Singapore

[CR71] Shafie H (2019) State onslaught and ethnic faultlines: oscillating identities of the Rohingya between Myanmar and Bangladesh. pp. 37–56

[CR72] Taylor L, Meissner F (2020). A crisis of opportunity: market-making, big data, and the consolidation of migration as risk. Antipode.

[CR73] Titifanue J, Varea RR, Varea R, Kant R, Finau G (2018). Digital diaspora, reinvigorating indigenous identity and online activism: social media and the reorientation of Rotuman identity. Media Int Aust.

[CR74] Tölölyan K (1991). The nation-state and its others: in lieu of a preface. Diaspora.

[CR75] Tonkin D (2014) The ‘Rohingya’ identity—British experience in Arakan 1826–1948. Network Myanmar, Rangoon

[CR76] Tsagarousianou R (2007) "Re-evaluating dispora": connectivity, mobilization and imagination in a globalised world. In: Sahoo AK, Maharaj B (ed.) Sociology of diaspora: a reader Jaipur, India Rawat. pp. 101-117

[CR77] Tsaliki L (2003) Globalisation and hybridity: the construction of Greekness on the internet. In: Karim K (ed.) The media of diaspora. Routledge, London

[CR78] Uddin N (2020). The Rohingya an ethnography of ‘Subhuman’ Life.

[CR79] Wade F (2017). Myanmar’s enemy within: Buddhist violence and the making of a muslim ‘other’.

[CR80] Whiteman N (2012). Undoing ethics rethinking practice in online research.

[CR81] Willis R (2017) Observations online: finding the ethical boundaries of Facebook research. Res Ethics 15(1):1–17

[CR82] Zuboff S (2019). The age of surveillance capitalism: the fight for a human future at the new frontier of power.

